# Bilateral thoracic paravertebral block for robotic hepatectomy without hepatic portal occlusion for a cirrhosis patient: a case report

**DOI:** 10.3389/fonc.2025.1684794

**Published:** 2026-01-12

**Authors:** Peng Ye, Chengyu Liao, Shi Chen, Fushan Xue, Xiaochun Zheng, Danfeng Wang

**Affiliations:** 1 Department of Anesthesiology, Shengli Clinical Medical College of Fujian Medical University, Fuzhou University Affiliated Provincial Hospital, Fuzhou, China; 2 Fujian Provincial Key Laboratory of Emergency Medicine, Fujian Provincial Key Laboratory of Critical Care Medicine, Fujian Provincial Co-Constructed Laboratory of “Belt and Road,” Fujian Emergency Medical Centre, Fuzhou, China; 3 Department of Hepatobiliary and Pancreatic Surgery, Shengli Clinical Medical College of Fujian Medical University, Fuzhou University Affiliated Provincial Hospital, Fuzhou, China

**Keywords:** intraoperative hemorrhage, low central venous pressure, robotic hepatectomy, surgical field visualization, thoracic paravertebral block

## Abstract

**Background:**

Bilateral thoracic paravertebral block (TPVB) is a regional anesthesia technique used for perioperative analgesia in thoracic and abdominal surgeries. However, its application as a key component of anesthesia to optimize surgical conditions during robotic-assisted hepatectomy, particularly in patients with severe liver cirrhosis, is not well-documented.

**Case presentation:**

We present the successful application of bilateral thoracic paravertebral block (TPVB) in conjunction with general anesthesia for robotic-assisted partial hepatectomy without hepatic portal occlusion in a 62-year-old female patient diagnosed with Child-Pugh class B liver cirrhosis. The procedure resulted in minimal intraoperative blood loss (80mL), stable hemodynamics under low central venous pressure, and an exceptionally clear surgical field, facilitating precise dissection. This procedure also markedly reduces sedative/analgesic requirements for general anesthesia and causes rapid emergence. The patient experienced effective postoperative analgesia with minimal opioid consumption and achieved early recovery milestones.

**Conclusions:**

This case highlights the potential of bilateral TPVB to reduce hepatic sinusoidal pressure and decrease catecholamine secretion through selective visceral sympathetic nerve block. Thereby minimizing intraoperative bleeding and enhancing surgical field visualization without the need for hepatic portal occlusion. It presents a viable anesthetic strategy to optimize surgical conditions in minimally invasive hepatobiliary procedures, especially for high-risk patients with limited physiological reserve.

## Background

Robotic-assisted hepatectomy has enhanced the precision of resecting tumors in anatomically challenging locations, such as the posterosuperior liver segments, owing to its three-dimensional magnified view, seven-degree-of-freedom instruments, and tremor filtration system (1, 2). Multicenter studies have demonstrated its superiority over conventional laparoscopy in terms of reduced blood loss and lower conversion-to-open rates, particularly in patients with hepatocellular carcinoma and underlying cirrhosis (3). However, parenchymal transection remains a challenge; oozing from the raw liver surface, exacerbated by elevated hepatic sinusoidal pressure, can impair surgical field clarity (4), which may increase the difficulty of the surgery and the operating costs.

The current standard of care involves general anesthesia combined with low central venous pressure (LCVP) management (CVP ≤5 cmH_2_O), primarily achieved through restrictive fluid administration and the use of vasoactive drugs to reduce hepatic venous pressure (5, 6). This approach, however, presents a dilemma. Strict fluid restriction is associated with multiple adverse outcomes (7), while the frequent use of vasopressors to maintain mean arterial pressure may compromise splanchnic perfusion and delay the return of bowel function (8). Meanwhile, robotic liver resection typically requires hepatic portal occlusion to reduce bleeding from the liver parenchymal transection. However, this may have adverse effects on postoperative liver function, particularly for patients with liver dysfunction and severe hepatic cirrhosis (9, 10). In addition, both the hepatic portal occlusion and the reduction in the metabolism of general anesthetics due to liver parenchyma resection can significantly impact the postoperative liver function of patients.

Thoracic Paravertebral Block (TPVB), as an important technique for perioperative analgesia in liver surgery, has shown significant advantages in reducing postoperative pain and improving the prognosis of patients in recent years (11). After hepatic resection, the reduction in liver volume leads to a decreased metabolic capacity for opioids. Paravertebral blockade can reduce the use of opioids during liver surgery (12). One important advantage of paravertebral blockade is its relatively minor impact on hemodynamics. Studies indicate that, compared to epidural blockade, paravertebral blockade demonstrates better performance in terms of hemodynamic stability, failure rate, and the risk of spinal hematoma. A stable hemodynamic state helps maintain appropriate central venous pressure, which is significant for reducing bleeding during liver surgeries (13).

Meanwhile, anatomical studies have revealed that hepatic sinusoidal pressure is directly regulated by sympathetic tone; activation of the T5-T10 sympathetic chain can increase sinusoidal pressure by mediating intrahepatic vasoconstriction via α1-adrenergic receptors (14).

Based on this, we hypothesized that TPVB could selectively block the hepatic sympathetic innervation, theoretically reducing hepatic sinusoidal pressure and minimizing bleeding from the liver parenchymal transection. This allows patients to avoid undergoing hepatic portal occlusion, thereby protecting postoperative liver function.

Here, we report the novel application of bilateral TPVB as an adjunct to general anesthesia in a patient undergoing robotic hepatectomy without hepatic portal occlusion. We aimed to assess the potential of this approach in minimizing intraoperative bleeding and opioid consumption to enhance surgical field clarity, maintain hemodynamic stability during low central venous pressure (LCVP) management, and facilitate improved recovery. A written informed consent was obtained from the patient to publish this case report and any accompanying images.

## Case presentation

A 62-year-old female (162 cm, 61 kg, details in **Table 1**) with a 2.0 cm × 1.4 cm tumor in segment VIII was scheduled for a robotic-assisted partial hepatectomy. Preoperative assessment revealed Child-Pugh class B liver cirrhosis with a MELD score of 10. The patient has a history of hypertension and diabetes for over 10 years; however, these conditions are currently well-controlled, and the American Society of Anesthesiologists (ASA) physical status classification is grade II.

**Table 1:** Baseline characteristics of the including patient.

Variables	Value
Age(years)	62
Sex	Female
Height(cm)	162
body weight(kg)	61
Total bilirubin(μmol/L)	28
Albumin(g/L)	32
INR	1.5
Platelets	90×10^9^/L
Ascites	Mild
Hepatic encephalopathy	None

**Figure 1:**
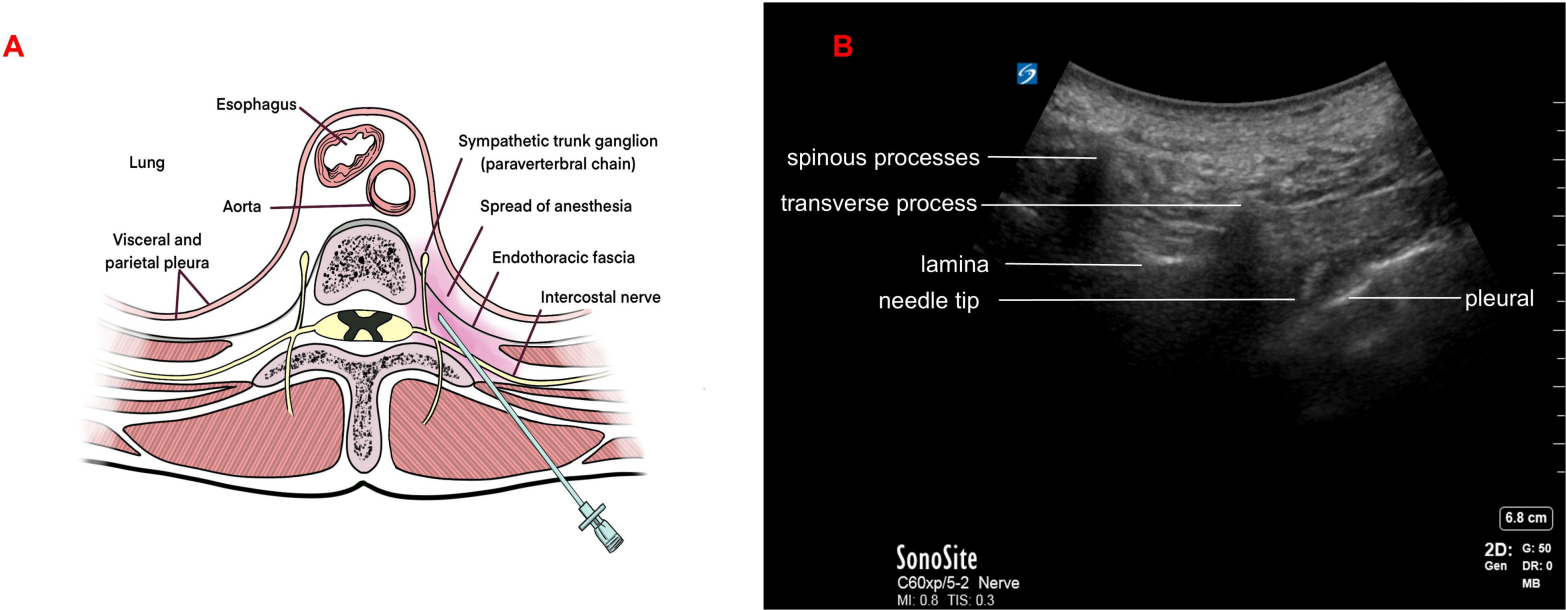
Bilateral thoracic paravertebral block. **(A)** Anatomical Diagram of Paravertebral Block. The thoracic paravertebral space is wedge-shaped, with the anterior-lateral side adjacent to the parietal pleura, and the posterior-medial side adjacent to the transverse processes and vertebral body. The costotransverse ligament forms the posterior wall of the space. **(B)** Ultrasound-guided thoracic paravertebral block was performed with punctures at the bilateral T7 and T10 Spaces respectively.

After establishing standard monitoring, bilateral TPVB was performed at the T7 and T10 levels under ultrasound guidance (**Figure 1**). At each of the four injection sites, 15 mL of 0.3% ropivacaine with epinephrine (5 µg/mL) was administered. Sensory blockade from T5 to T12 was confirmed 30 minutes later, evaluated using a cold glass vial stored at 5°C. General anesthesia was induced with sufentanil 0.3 μg/kg, propofol via target-controlled infusion (effect-site concentration 3 µg/mL) and rocuronium bromide 0.6 mg/kg. Anesthesia was maintained with sevoflurane (MAC 0.8–1.0) and remifentanil (0.05–0.1 µg/kg/min).The bispectral index (BIS) was maintained between 40 and 60.

**Figure 2:**
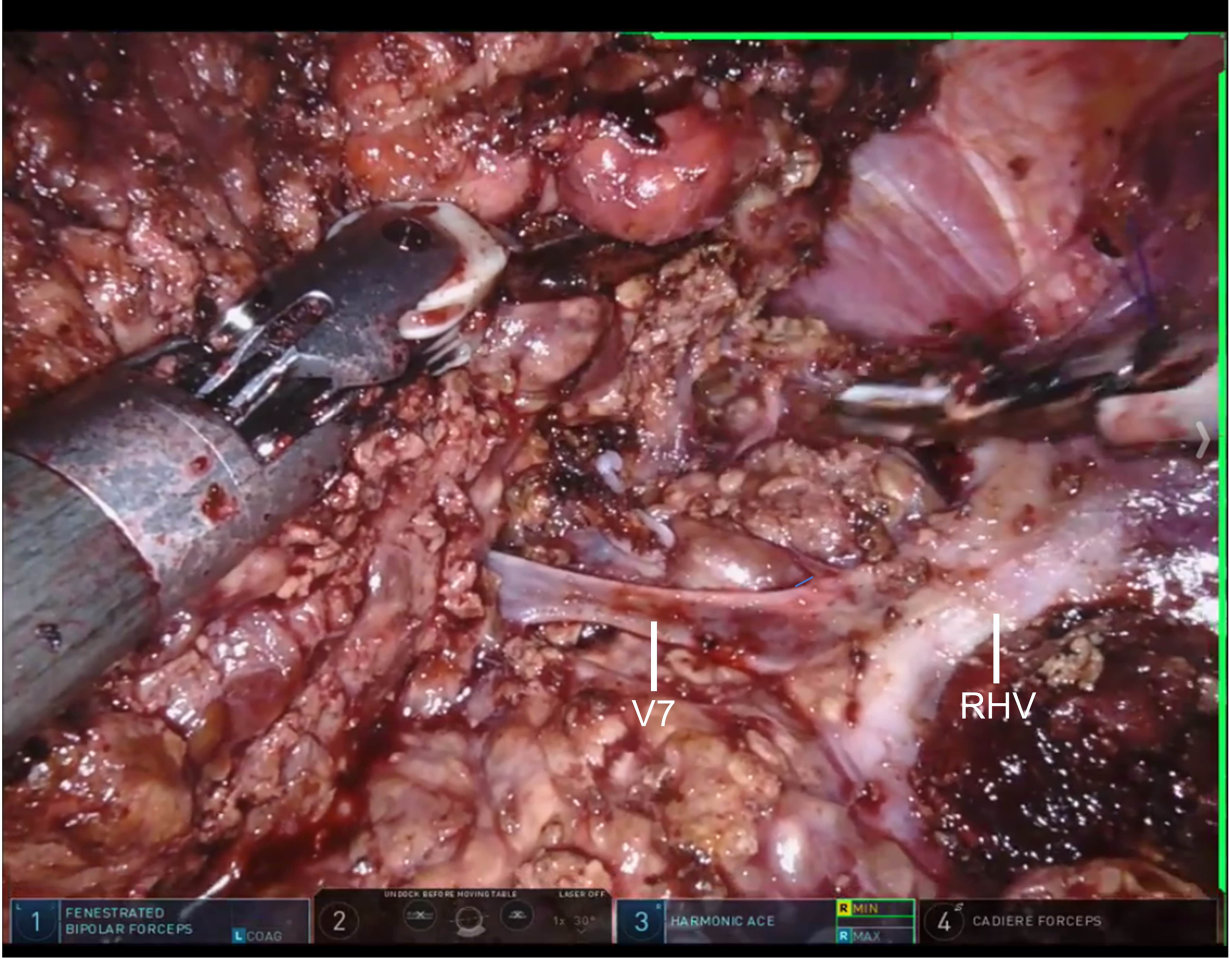
DaVinci robotic-assisted laparoscopic resection of hepatocellular carcinoma without hepatic portal occlusion. On hepatic cross-sectional imaging, the tumor is clearly delineated. The surgical field is free of significant bleeding, and the right hepatic vein is collapsed. RHV:Right Hepatic Vein; V7: Right Hepatic Vein, RHV.

Intraoperatively, LCVP was maintained at ≤5 cmH_2_O via a right internal jugular vein catheter, with stroke volume variation (SVV) kept between 15% and 23%. Continuous arterial pressure was monitored via a radial artery catheter, and norepinephrine was used to maintain mean arterial pressure within 20% of the baseline value. The robotic system was docked in 8 minutes. Liver parenchymal transection was performed using a combination of the CUSA system and bipolar coagulation without a Pringle maneuver. Segment VIII, along with its Glissonian pedicle, was completely resected along the middle and right hepatic veins without hepatic portal occlusion (**Figure 2**). The total surgical time was 190 minutes, with the parenchymal transection phase lasting 48 minutes. Total blood loss, quantified by a combination of suction canister weighing and soaked gauze evaluation, was 80 mL. The surgical field clarity was independently rated as Grade 0–1 by two surgeons, who were blinded to the anesthetic technique, using the Validated Intraoperative Bleeding (VIBe) Scale based on surgical video recordings. According to the VIBe Scale, Grade 0 indicates no bleeding, while Grade 1 indicates mild, oozing/intermittent bleeding that does not interfere with surgical progress, corresponding to excellent surgical field clarity (15).

The patient was extubated 10 minutes post-surgery. Postoperative pain was managed with a patient-controlled intravenous analgesia (PCIA) pump containing sufentanil (2 µg/kg) and toisetron (10 mg) in 100 mL of 0.9% saline. The pump was programmed with no background infusion, a bolus dose of 2.0 mL, and a lockout interval of 15 minutes. The patient received no analgesic agent during the first 6 h post-surgery. The first 24-hour oral morphine equivalent consumption was 30 mg, with numeric rating scale (NRS) pain scores of 2 at rest and 4 during movement in POD1. The patient ambulated with assistance at 6 hours postoperatively and passed flatus within 24 hours. On the first postoperative day, the alanine aminotransferase level of the patient rose to 110 U/L, and it returned to normal levels on the third postoperative day.No bleeding, infection, or liver decompensation occurred during the 30-day follow-up period.

## Discussion

To our knowledge, this is the first case report describing the use of bilateral TPVB in robotic-assisted hepatectomy, demonstrating its potential to not only provide effective analgesia but also to significantly reduce intraoperative bleeding and improve surgical field clarity.

In terms of analgesia and enhanced recovery, the patient’s postoperative opioid consumption was minimal (30 mg oral morphine equivalent in first 24 hours). This is likely because bilateral TPVB provides dual somatic and visceral pain control by blocking both the posterior rami of the spinal nerves (somatic pain) and the visceral sympathetic fibers (11, 16). Unlike thoracic epidural analgesia, which often requires limited local anesthetic volumes to avoid motor block and hypotension, TPVB offers profound analgesia with superior hemodynamic stability. This stability was crucial for maintaining the target LCVP with minimal vasopressor support, thus preserving gut perfusion and contributing to the patient’s early return of bowel function and mobilization, which are core tenets of Enhanced Recovery After Surgery (ERAS) protocols (17, 18). Although this report did not identify any complications related to TPVB, it is important to note that when performing TPVB in patients with abnormal liver function or concurrent coagulation disorders, particular vigilance is required to prevent the occurrence of paravertebral hematoma.

Currently, hepatic portal occlusion has gradually become a common surgical procedure in laparoscopic and robot-assisted liver resection. Its primary purpose is to reduce bleeding during the liver resection process. However, hepatic portal occlusion not only increases ischemia-reperfusion injury to liver cells, especially in patients with liver cirrhosis who are prone to liver failure after surgery, but also raises the postoperative tumor recurrence rate and decreases patient survival (19). This case report successfully utilized bilateral TPVB as an auxiliary anesthesia method during robot-assisted liver cancer resection in a patient with severe liver cirrhosis, reducing intraoperative blood loss without performing hepatic portal occlusion.The observed reduction in blood loss to 80 mL, well below the reported mean for robotic hepatectomy. Thoracic paravertebral block (TPVB) confers hemostatic benefits in liver resections may by synergistically modulating sympathetic outflow, central venous pressure (CVP), and stress-induced catecholamine release. First, by targeting T5–T10 sympathetic fibers (11), TPVB attenuates visceral vascular smooth muscle contraction, thereby decreasing local vascular resistance and perfusion pressure at the resection margin, which translates into reduced surgical bleeding. Second, when combined intraoperatively with judicious fluid restriction and slight positional adjustments to maintain a low CVP, TPVB further diminishes hepatic venous return pressure, curtailing back-bleeding from transected sinusoids (20, 21). Finally, the superior analgesia and sedation achieved with TPVB mitigate perioperative nociceptive stimuli and blunt stress-related catecholamine surges, reducing intraoperative hypertension and secondary vasodilatory “passive” bleeding. Collectively, these mechanisms establish TPVB as a valuable adjunct for minimizing blood loss and stabilizing hemodynamics during hepatic surgery. This effect is particularly advantageous in robotic surgery, where the magnified three-dimensional view is highly sensitive to even minor oozing, and a clear field allows for more precise dissection and potentially shorter transection times.

This study has limitations. As this is a single case report, its findings are not generalizable. Furthermore, the absence of direct measurement of hepatic sinusoidal pressure, a theoretical mechanism that was inferred but not validated in this study, represents a limitation. Therefore, high-quality randomized controlled trials are urgently needed to determine whether bilateral paravertebral blockade can improve hepatic bleeding during liver surgery performed with the assistance of the da Vinci robotic system.The successful implementation of ultrasound-guided bilateral TPVB requires a significant learning curve to minimize the risk of complications such as pneumothorax. Furthermore, the pharmacokinetics of ropivacaine may be altered in patients with liver cirrhosis, necessitating careful consideration of drug dosage and concentration.

## Conclusion

This case demonstrates that the use of bilateral thoracic PVB as an adjunct to general anesthesia appears to be a promising technique for robotic-assisted hepatectomy. It can reduce intraoperative bleeding and create an excellent surgical field, with an excellent postoperative pain control and a rapid postoperative function recovery. However, further randomized controlled trials are warranted to determine these potential benefits of bilateral thoracic PVB for robotic-assisted hepatectomy.

## Data Availability

The raw data supporting the conclusions of this article will be made available by the authors, without undue reservation.

## References

[1] Finotti M D’amico F Mulligan D Testa G . A narrative review of the current and future role of robotic surgery in liver surgery and transplantation. Hepatobiliary Surg Nutr. (2021) 12:56–68. doi: 10.21037/hbsn-21-115, PMID: 36860258 PMC9944521

[2] Rouault A Richa Y Truant S . National impact and advantages of the robotic approach to liver surgery in the era of minimally invasive surgery. Hepatobiliary Surg Nutr. (2025) 14:275–8. doi: 10.21037/hbsn-2025-94, PMID: 40342778 PMC12057515

[3] Pilz Da Cunha G De Meyere C D'hondt M Swijnenburg RJ . Robotic liver parenchymal transection using the SynchroSeal. Surg Endosc. (2024) 38:4947–55. doi: 10.1007/s00464-024-11005-4, PMID: 38977499 PMC11362496

[4] Romano F Garancini M Uggeri F Degrate L Nespoli L Gionotti L . Bleeding in hepatic surgery: sorting through methods to prevent it. HPB Surg. (2012) 2012:169351. doi: 10.1155/2012/169351, PMID: 23213268 PMC3506885

[5] Yu L Sun H Jin H Tan H . The effect of low central venous pressure on hepatic surgical field bleeding and serum lactate in patients undergoing partial hepatectomy: a prospective randomized controlled trial. BMC Surg. (2020) 20:25. doi: 10.1186/s12893-020-0689-z, PMID: 32019557 PMC7001244

[6] Coeckelenbergh S Soucy-Proulx M van der Linden P Roullet S Moussa M Kato H . Restrictive versus Decision Support Guided Fluid Therapy during Major Hepatic Resection Surgery: A Randomized Controlled Trial. Anesthesiology. (2024) 141:881–90. doi: 10.1097/ALN.0000000000005175, PMID: 39052844 PMC12713225

[7] Hoeter K Heinrich S Wollschläger D Melchior F Noack A Tripke V . The optimal fluid strategy matters in liver surgery: A retrospective single centre analysis of 666 consecutive liver resections. J Clin Med. (2023) 12. doi: 10.3390/jcm12123962, PMID: 37373656 PMC10299667

[8] Jozwiak M Geri G Laghlam D Boussion K Dolladille C Nguyen LS . Vasopressors and risk of acute mesenteric ischemia: A worldwide pharmacovigilance analysis and comprehensive literature review. Front Med (Lausanne). (2022) 9:826446. doi: 10.3389/fmed.2022.826446, PMID: 35677822 PMC9168038

[9] Huang XK Lu WF Liu SY Fu TW Jin L Du CF . Multicenter propensity score-matched analysis to compare perioperative morbidity after laparoscopic or robotic complex hepatectomy for solitary hepatocellular carcinoma. HPB (Oxford). (2024) 26:1062–71. doi: 10.1016/j.hpb.2024.05.013, PMID: 38830783

[10] Ratti F Marino R Aldrighetti L . Improving performance of robotic liver resections with high technical complexity by Robo-Lap approach. Hepatobiliary Surg Nutr. (2023) 12:981–6. doi: 10.21037/hbsn-23-378, PMID: 38115920 PMC10727833

[11] Chen H Liao Z Fang Y Niu B Chen A Cao F . Continuous right thoracic paravertebral block following bolus initiation reduced postoperative pain after right-lobe hepatectomy: a randomized, double-blind, placebo-controlled trial. Reg Anesth Pain Med. (2014) 39:506–12. doi: 10.1097/AAP.0000000000000167, PMID: 25304475 PMC4218764

[12] Dudek P Zawadka M Andruszkiewicz P Gelo R Pugliese F Bilotta F . Postoperative analgesia after open liver surgery: systematic review of clinical evidence. J Clin Med. (2021) 10. doi: 10.3390/jcm10163662, PMID: 34441958 PMC8397227

[13] Mcnally SJ Revie EJ Massie LJ McKeown DW Parks RW Garden OJ . Factors in perioperative care that determine blood loss in liver surgery. HPB (Oxford). (2012) 14:236–41. doi: 10.1111/j.1477-2574.2011.00433.x, PMID: 22404261 PMC3371209

[14] Jensen KJ Alpini G Glaser S . Hepatic nervous system and neurobiology of the liver. Compr Physiol. (2013) 3:655–65. doi: 10.1002/j.2040-4603.2013.tb00506.x PMC3733049 23720325

[15] Aparicio-López D Asencio-Pascual JM Blanco-Fernández G Cugat-Andorrà E Gómez-Bravo MA López-Ben S . Evaluation of the validated intraoperative bleeding scale in liver surgery: study protocol for a multicenter prospective study. Front Surg. (2023) 10:1223225. doi: 10.3389/fsurg.2023.1223225, PMID: 37850041 PMC10577188

[16] Lee JH Kim CS Kim H Choi JM Kim Y Jeong SM . Preemptive visceral analgesic effect of thoracic paravertebral block on postoperative opioid consumption in patients undergoing laparoscopic cholecystectomy: a prospective, randomized, assessor-blind study. Korean J Anesthesiol. (2023) 76:203–12. doi: 10.4097/kja.22481, PMID: 36539924 PMC10244605

[17] Oehring R Keshi E Hillebrandt KH Koch PF Felsenstein M Moosburner S . Neudecker J et al: Enhanced recovery after surgery society's recommendations for liver surgery reduces non surgical complications. Sci Rep. (2025) 15:3693. doi: 10.1038/s41598-025-86808-z, PMID: 39880966 PMC11779921

[18] Téoule P Dunker N Debatin J Sturm D Hetjens S Walter V . Reduction of central venous pressure in elective robotic and laparoscopic liver resection: the PRESSURE trial-A randomized clinical study. Ann Surg. (2025) 282:210–8. doi: 10.1097/SLA.0000000000006721, PMID: 40197483

[19] Tang SC Diao YK Lin KY Li C Xu X Liang L . Association of Pringle maneuver with postoperative recurrence and survival following hepatectomy for hepatocellular carcinoma: a multicenter propensity score and competing-risks regression analysis. Hepatobiliary Surg Nutr. (2024) 13:412–24. doi: 10.21037/hbsn-23-7, PMID: 38911192 PMC11190521

[20] Bolognesi M Di Pascoli M Verardo A Gatta A . Splanchnic vasodilation and hyperdynamic circulatory syndrome in cirrhosis. World J Gastroenterol. (2014) 20:2555–63. doi: 10.3748/wjg.v20.i10.2555, PMID: 24627591 PMC3949264

[21] Li Y Zhu B Shi K Lu Y Zeng X Li Y . Advances in intrahepatic and extrahepatic vascular dysregulations in cirrhotic portal hypertension. Front Med (Lausanne). (2025) 12:1515400. doi: 10.3389/fmed.2025.1515400, PMID: 39958826 PMC11825794

